# Human mesenchymal stem cells ameliorate experimental pulmonary hypertension induced by maternal inflammation and neonatal hyperoxia in rats

**DOI:** 10.18632/oncotarget.19388

**Published:** 2017-07-19

**Authors:** Chung-Ming Chen, Willie Lin, Liang-Ti Huang, Hsiu-Chu Chou

**Affiliations:** ^1^ Department of Pediatrics, Taipei Medical University Hospital, Taipei, Taiwan; ^2^ Department of Pediatrics, School of Medicine, College of Medicine, Taipei Medical University, Taipei, Taiwan; ^3^ Meridigen Biotech Co., Ltd., Taipei, Taiwan; ^4^ Department of Pediatrics, Wan Fang Hospital, Taipei Medical University, Taipei, Taiwan; ^5^ Department of Anatomy and Cell Biology, School of Medicine, College of Medicine, Taipei Medical University, Taipei, Taiwan

**Keywords:** lipopolysaccharide, hyperoxia, pulmonary hypertension, β-myosin heavy chain, toll-like receptor 4

## Abstract

Pulmonary hypertension is a critical problem in infants with bronchopulmonary dysplasia. This study determined the therapeutic effects of human mesenchymal stem cells (MSCs) on pulmonary hypertension in an animal model. Pregnant Sprague–Dawley rats were intraperitoneally injected with lipopolysaccharide (LPS, 0.5 mg/kg/day) on gestational days 20 and 21. The pups were randomly assigned to two treatment conditions: room air (RA) or an O_2_-enriched atmosphere. On postnatal day 5, they were intratracheally transplanted with human MSCs (3 × 10^5^ and 1 × 10^6^ cells) in 0.03 mL of normal saline (NS). Five study groups were examined: normal, LPS+RA+NS, LPS+O_2_+NS, LPS+O_2_+MSCs (3 × 10^5^ cells), and LPS+O_2_+MSCs (1 × 10^6^ cells). On postnatal day 14, the pup lungs and hearts were collected for histological examinations. The LPS+RA+NS and LPS+O_2_+NS groups exhibited a significantly higher right ventricle (RV):left ventricle (LV) thickness ratio and medial wall thickness (MWT) and higher β-myosin heavy chain (β-MHC) and toll-like receptor (TLR) 4 expression than did the normal group. Human MSC transplantation in LPS- and O_2_-treated rats reduced the MWT, RV:LV thickness ratio, and β-MHC and TLR4 expression to normal levels. Thus, intratracheal human MSC transplantation ameliorates pulmonary hypertension, probably by suppressing TLR4 expression in newborn rats.

## INTRODUCTION

Bronchopulmonary dysplasia (BPD), a severe and common chronic lung disease in infants, is caused by interrupted alveolar and vascular growth [1, 2]. Pulmonary hypertension was reported in 37% of extremely low-birth-weight infants with severe BPD [3]. Pulmonary hypertension complicates the course of BPD and is associated with considerable mortality and morbidity [4–7]. Similar to human infants with pulmonary hypertension and BPD, rat pups exposed to hyperoxia exhibit pulmonary hypertension, right ventricular hypertrophy, pulmonary vascular remodeling, and alveolar simplification [8]. The pathogenesis of pulmonary hypertension in BPD is complex and may result from interactions between antenatal risk factors, such as pregnancy-induced hypertension, intrauterine growth restriction, and infection, and postnatal risk factors, such as oxidative stress and inflammation, in preterm infants with underlying genetic susceptibility [9]. However, the most effective treatment for BPD-associated pulmonary hypertension remains unclear.

Mesenchymal stem cells (MSCs) are multipotent stromal cells characterized by the ability to self-renew and differentiate into various cell types including bone, cartilage, adipose tissue, muscle, and tendons [10]. Prenatal inflammation and postnatal hyperoxia were to reduce pulmonary vascular density and induce right ventricular hypertrophy and pulmonary hypertension in newborn rats [11]. Preclinical studies have demonstrated the efficacy of MSCs or MSC-conditioned medium in alleviating hyperoxia-induced BPD and pulmonary hypertension [12–14]. Nevertheless, the effects of MSCs on pulmonary hypertension induced by maternal inflammation and neonatal hyperoxia remain unknown. We hypothesized that the intratracheal administration of human MSCs ameliorates experimental pulmonary hypertension in newborn rats. This study explored the effects of maternal inflammation and neonatal hyperoxia on heart morphology and the therapeutic effects of human MSCs on pulmonary hypertension in rat pups.

## RESULTS

### Changes in body and heart weights and heart:body weight ratio

On postnatal day 14, the body and heart weights and heart:body weight ratio were comparable between normal rats and rats treated with normal saline (NS) or MSC (Table 1).

**Table 1:** Body and heart weights and heart:body weight and RV:LV ratios in rat pups on postnatal day 14

Treatment	*n*	Body weight (g)	Heart weight (g)	Heart:body weight (%)	RV:LV
Normal	17	25.4 ± 2.4	0.16 ± 0.02	0.63 ± 0.05	0.33 ± 0.10
LPS+RA+NS	10	24.2 ± 2.6	0.15 ± 0.04	0.62 ± 0.07	0.53 ± 0.15
LPS+ O_2_+NS	14	20.7 ± 5.8	0.14 ± 0.04	0.66 ± 0.06	0.87±0.22**^,^***
LPS+O_2_+MSCs (3 × 10^5^ cells)	6	23.1 ± 7.1	0.14 ± 0.05	0.62 ± 0.05	0.61 ± 0.18*
LPS+O_2_+MSCs (1 × 10^6^ cells)	7	20.2 ± 1.2	0.13 ± 0.01	0.66 ± 0.06	0.56 ± 0.20*

Values are expressed as the mean ± standard deviation.

*P < 0.05 vs. LPS+O_2_+NS group, **P < 0.01 vs. LPS+RA+NS group, and ***P < 0.001 vs. normal group.

### Right ventricular hypertrophy and pulmonary arterial remodeling

**Figure 1:**
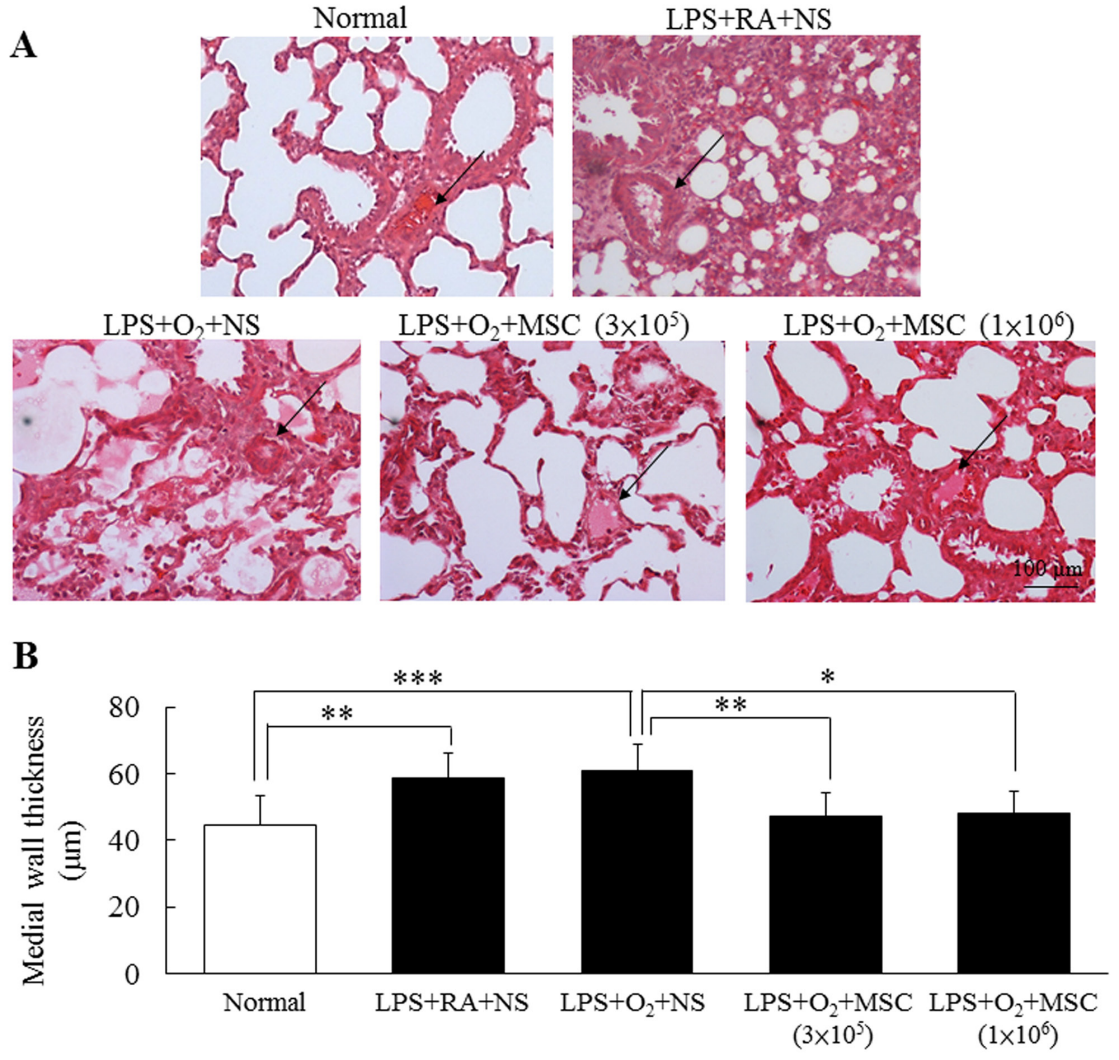
**(A)** Representative lung sections stained with hematoxylin and eosin and **(B)** MWT in 14-day-old prenatal LPS- and neonatal hyperoxia-treated rats and rats that received NS or human MSC treatment on postnatal day 5. Prenatal LPS and neonatal hyperoxia treatment significantly increased the MWT in the rats on postnatal day 14. Compared with the NS-treated rats, the MSC-treated rats exhibited a significantly decreased MWT on postnatal day 14 (*P < 0.05, **P < 0.01, and *P* < 0.001). Arrows indicate blood vessels.

We calculated the right ventricle (RV):left ventricle (LV) thickness ratio as an indicator of right ventricular hypertrophy. The rats exposed to prenatal lipopolysaccharide (LPS) and neonatal hyperoxia and treated with NS had a significantly higher RV:LV thickness ratio than did the normal rats and those exposed to prenatal LPS (Table 1). Human MSC transplantation (3 × 10^5^ and 1 × 10^6^ cells) in the prenatal LPS- and neonatal hyperoxia-treated rats significantly reduced the RV:LV thickness ratio to that reported in the LPS+room air (RA)+NS group. In addition, we estimated arterial remodeling by measuring the medial wall thickness (MWT) of the small pulmonary arteries, because the MWT is a surrogate marker of pulmonary hypertension. The rats exposed to prenatal LPS and reared in either RA or an O_2_-enriched atmosphere exhibited a significantly higher MWT than did the normal rats. Treatment with human MSCs (3 × 10^5^ and 1 × 10^6^ cells) significantly reduced the prenatal LPS- and neonatal hyperoxia-induced increase in MWT (Figure 1).

### Immunohistochemistry for β-myosin heavy chain

**Figure 2:**
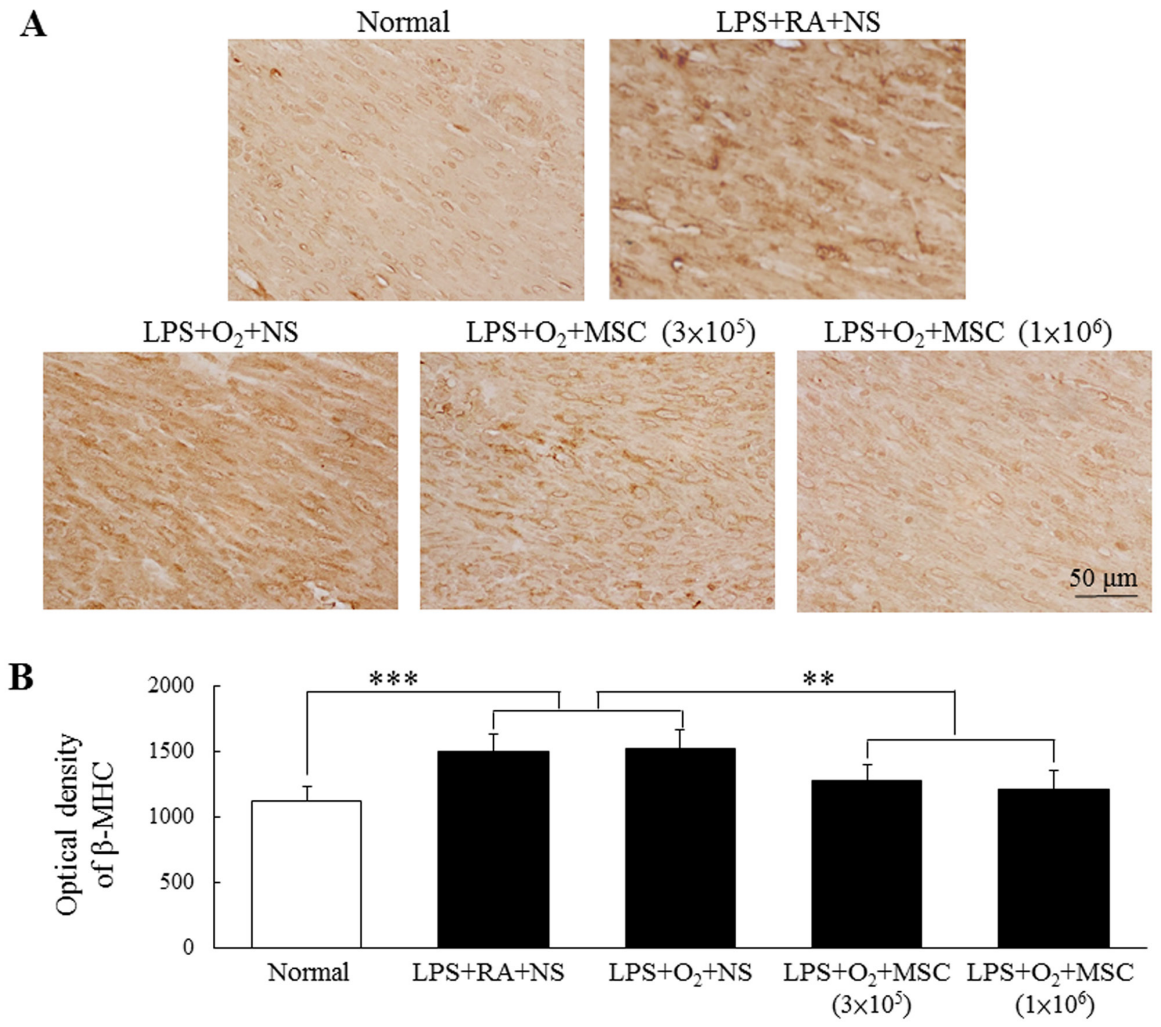
**(A)** Immunohistochemistry of β-MHC in heart sections and **(B)** semiquantitative analysis of β-MHC expression on postnatal day 14**.** Positive staining is depicted in brown. β-MHC was mainly distributed in the myocardial cell cytoplasm. Prenatal LPS and neonatal hyperoxia treatment increased β-MHC expression in the rats. β-MHC expression was significantly reduced in the human MSC-treated rats compared with the NS-treated rats (**P < 0.01 and ***P < 0.001).

β-myosin heavy chain (β-MHC) expression was primarily detected in the myocardial cell cytoplasm (Figure 2A). β-MHC expression was significantly higher in the rats exposed to prenatal LPS and neonatal hyperoxia than in the normal rats. A semiquantitative analysis revealed increased β-MHC expression in the rats exposed to prenatal LPS and reared in either RA or O_2_ (Figure 2B). Treatment with human MSCs (3 × 10^5^ and 1 × 10^6^ cells) significantly reduced β-MHC expression in the prenatal LPS- and neonatal hyperoxia-treated rats compared with that in the normal rats.

### Immunohistochemistry for toll-like receptor 4

**Figure 3:**
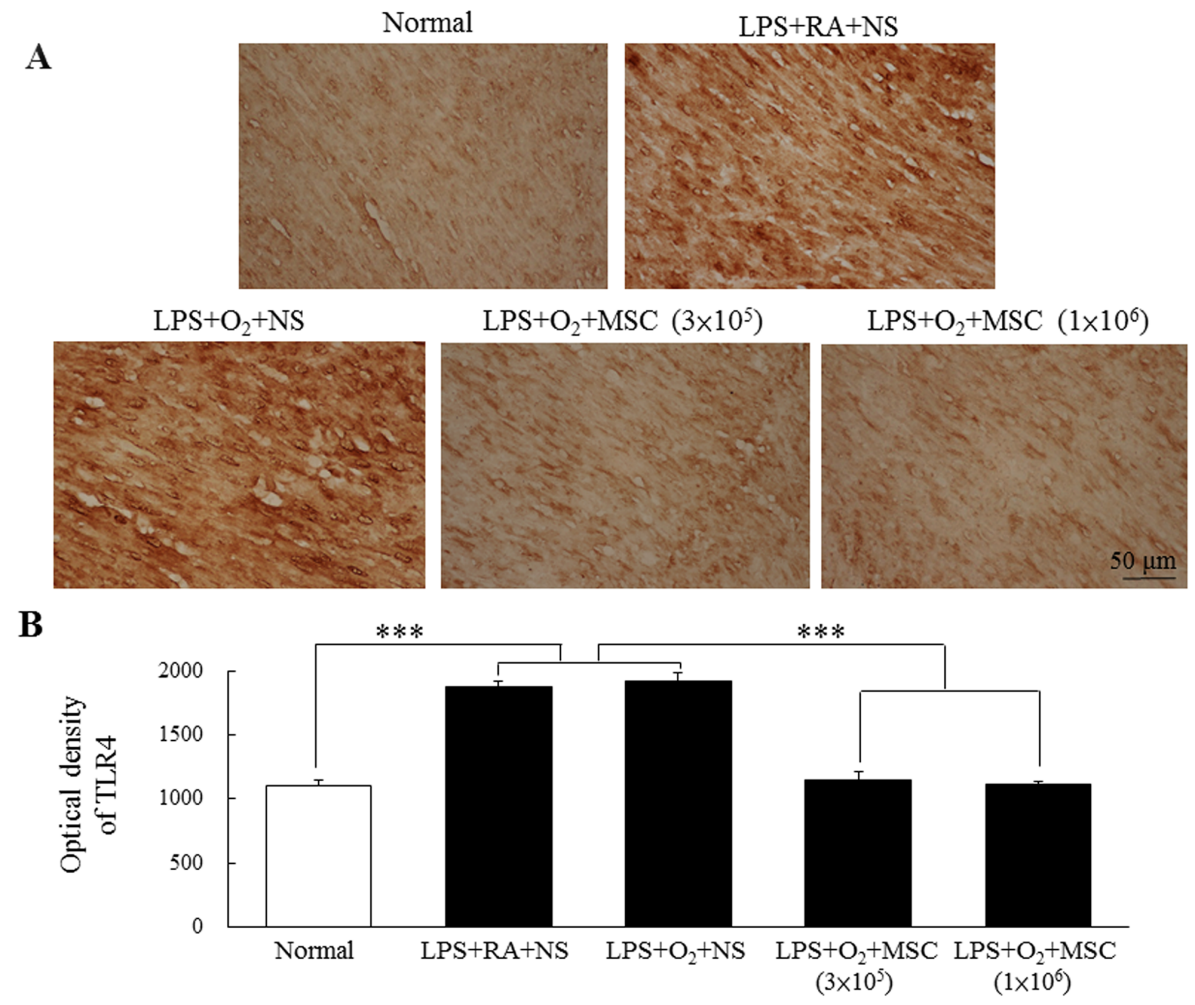
**(A)** Immunohistochemistry of TLR4 in heart sections and **(B)** semiquantitative analysis of TLR4 expression on postnatal day 14. Positive staining is shown in brown. TLR4 expression was diffused and predominantly confined to cardiomyocytes. The rats exposed to prenatal LPS and reared in either RA or O_2_ exhibited increased TLR4 expression. Treatment with human MSCs (3 × 10^5^ and 1 × 10^6^ cells) significantly reduced TLR4 expression in the prenatal LPS- and neonatal hyperoxia-treated rats compared with the normal rats (***P < 0.001).

The prenatal LPS-treated rats exhibited higher toll-like receptor (TLR) 4 expression than did the normal rats (Figure 3A). A semiquantitative analysis revealed increased TLR4 expression in the rats exposed to prenatal LPS and reared in either RA or O_2_ (Figure 3B). Treatment with human MSCs (3 × 10^5^ and 1 × 10^6^ cells) significantly reduced this expression in the prenatal LPS- and neonatal hyperoxia-treated rats compared with that in the normal rats.

### Western blotting analysis for β-MHC and TLR4

**Figure 4:**
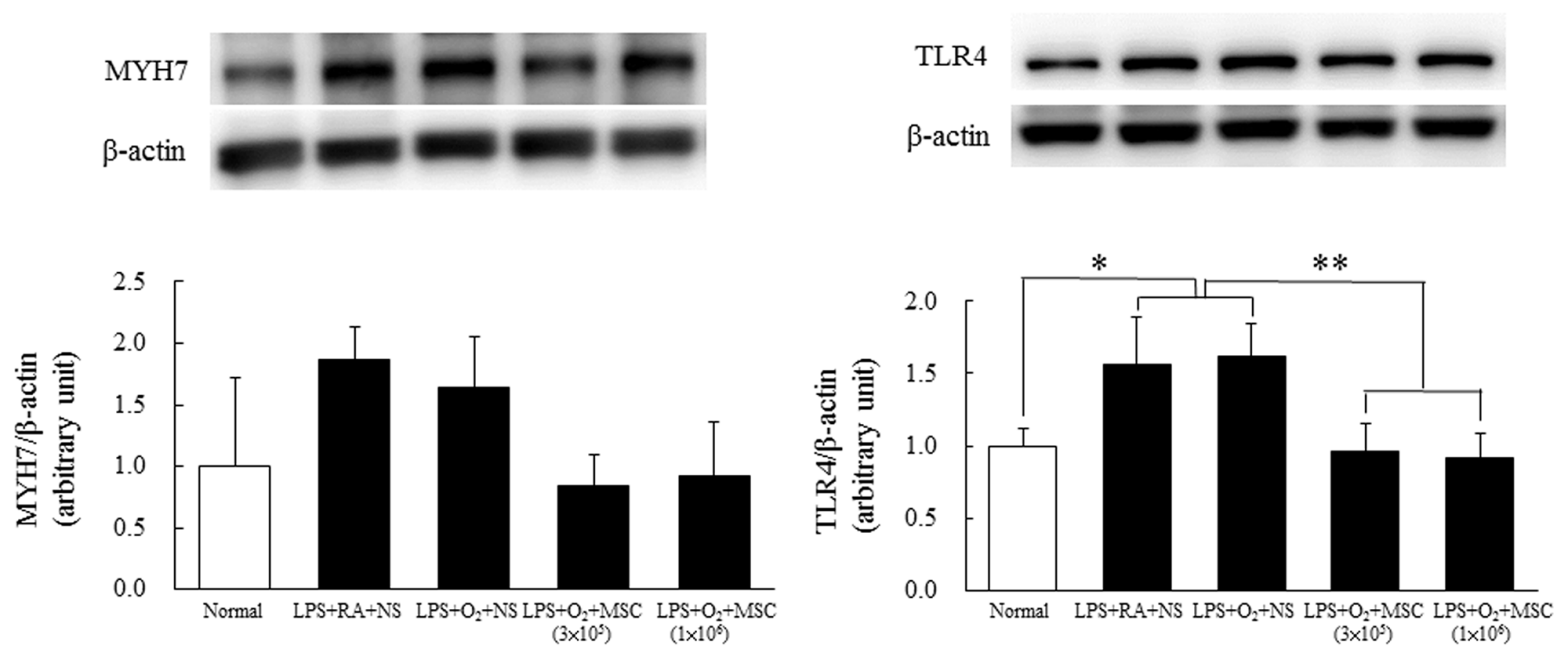
Representative Western blots and quantitative data determined using densitometry for β-MHC **(A)** and TLR4 **(B)** expression in heart tissues on postnatal day 14**.** The rats exposed to prenatal LPS and reared in either RA or O_2_ exhibited increased β-MHC and TLR4 expression. TLR4 expression was significantly reduced in the rats treated with MSCs (3 × 10^5^ and 1 × 10^6^ cells) compared with those treated with NS (*P < 0.05 and **P < 0.01). The difference was statistically nonsignificant for β-MHC.

Figure 4 shows increased β-MHC and TLR4 expression levels in the heart tissues of the rats exposed to prenatal LPS and reared in either RA or O_2_. Treatment with human MSCs (3 × 10^5^ and 1 × 10^6^ cells) significantly reduced TLR4 expression in the prenatal LPS- and neonatal hyperoxia-treated rats compared with that in the NS-treated rats; the difference was statistically nonsignificant for β-MHC.

### Detection of human MSCs in lung tissues

**Figure 5:**
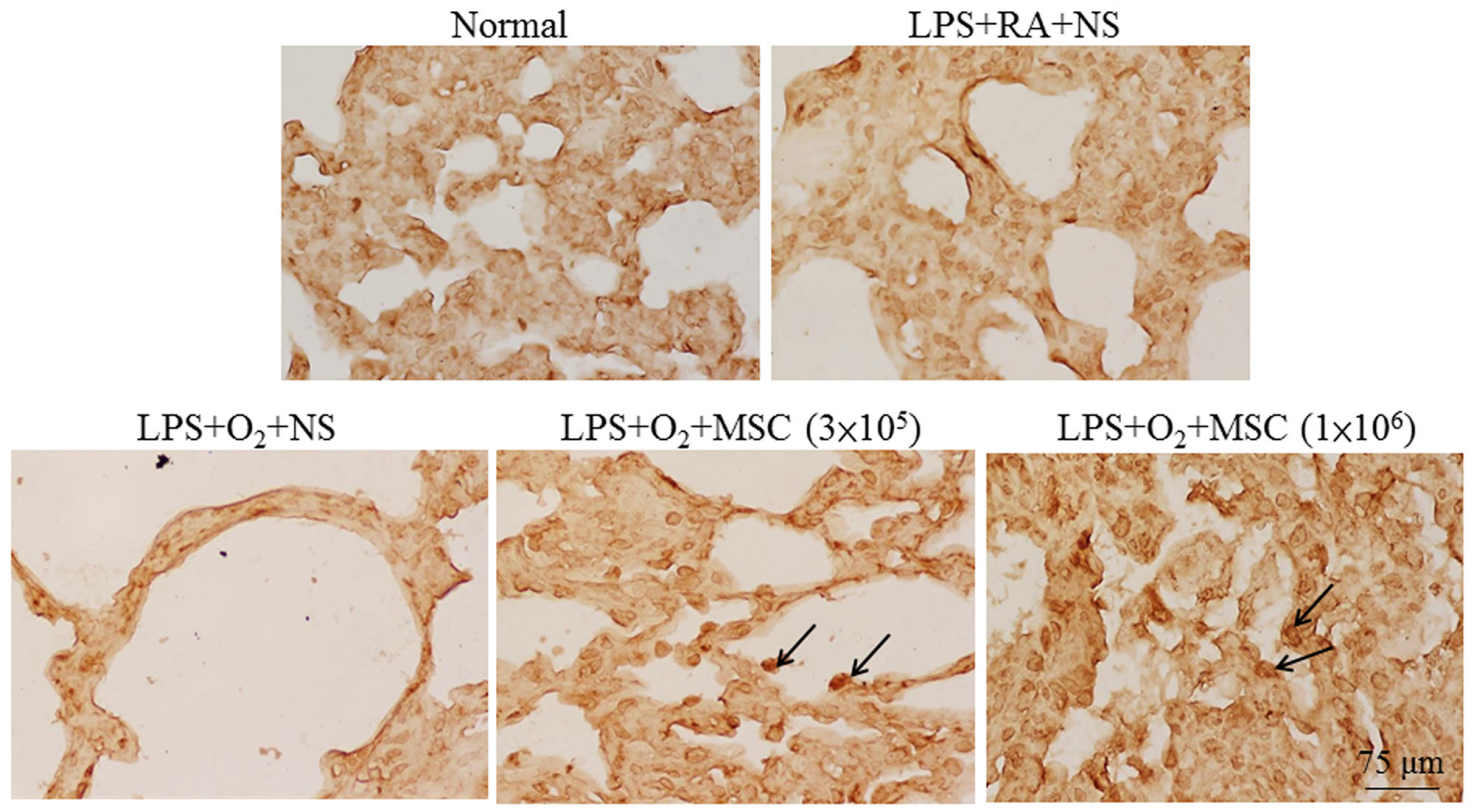
Representative immunohistochemical staining for a human-specific nuclear antigen in lung tissues on postnatal day 14 Positive staining is depicted in brown on the nuclei of MSCs (arrows).

To detect human MSCs in transplanted rat lungs, we used a human nuclear antigen-specific antibody. As shown in Figure 5, human-originated cells were detected in rat lung tissues treated with human MSCs.

## DISCUSSION

The main findings of this *in vivo* study are that the prenatal LPS and neonatal hyperoxia intervention increased the pulmonary arterial MWT, RV:LV thickness ratio, and β-MHC and TLR4 expression in rat hearts on postnatal day 14. However, the intratracheal administration of MSCs on postnatal day 5 reduced these parameters. These data suggest that human MSCs ameliorated right ventricular hypertrophy and pulmonary hypertension in the prenatal LPS- and neonatal hyperoxia-treated rats by suppressing TLR4 expression through paracrine effects.

Maternal, genetic, and environmental factors lead to an early injury of the developing lung, which impairs angiogenesis and alveolarization, thus resulting in the simplification of the distal lung airspace and clinical manifestations of BPD. Impairments in the developing pulmonary vasculature may cause severe pulmonary vascular disease; this disease in preterm infants with BPD is characterized by altered lung vascular development, growth, structure, or function, which precedes the onset of measurable pulmonary hypertension [9]. Pulmonary hypertension caused by pulmonary arterial remodeling and the disruption of angiogenic factor expression has been reported in the autopsy data of infants who died because of BPD [15, 16]. Recent animal studies have shown that intervention through either prenatal inflammation or postnatal hyperoxia could cause pulmonary hypertension in newborn rats [17, 18]. However, the treatment for pulmonary hypertension induced by prenatal inflammation and postnatal hyperoxia remains unclear.

Sammour et al. isolated MSCs from the bone marrow of adult male and female Sprague–Dawley rats and observed that female-derived MSCs have greater therapeutic efficacy than male MSCs in reducing neonatal hyperoxia-induced lung inflammation [19]. Studies have not investigated the sex effects of placental MSCs on hyperoxia-induced lung injury. In this study, human MSCs were derived from the placental tissues of women who had delivered male babies. The placental MSCs were of maternal origin, despite the babies being of the male sex. The sex effects of MSCs on hyperoxia-induced lung injury could not be determined in this study.

Markers of pulmonary hypertension, including indices of right ventricular hypertrophy, are RV/LV + septum weight, RV/LV + septum thickness ratio, and RV:LV thickness ratio [20–22]. Wagenaar et al. used the RV:LV thickness ratio to determine right ventricular hypertrophy in neonatal hyperoxia-induced lung injury [21]. Jone et al. used the RV/LV end-systolic diameter for evaluating outcomes in children with pulmonary hypertension [22]. In the current study, we used the RV:LV thickness ratio, pulmonary arterial MWT, and β-MHC expression as the indicators of right ventricular hypertrophy, pulmonary hypertension, and cardiac hypertrophy, respectively. We observed that the rats exposed to prenatal LPS, reared in either RA or O_2_, and treated with NS exhibited significantly higher markers of the aforementioned conditions than did the normal rats. The rats exposed to prenatal LPS and reared in O_2_ did not exhibit a significantly higher pulmonary arterial MWT or β-MHC level than those of the rats exposed to prenatal LPS and reared in RA. These results suggest that neonatal hyperoxia exposure does not exacerbate prenatal LPS-induced pulmonary hypertension on postnatal day 14.

LPS, a pathogen-associated molecule, is present in the outer membrane of most gram-negative bacteria. LPS was reported to activate TLR4 and downstream TLR signaling molecule expression in bovine endometrial epithelial cells [23]. TLRs are essential components of the innate immune system that recognize pathogens and initiate inflammatory responses [24]. LPS induced an increase in myocardial tumor necrosis factor (TNF)-α and interleukin-1β mRNA expression, and the response was blunted in TLR4-deficient mice [25]. We previously demonstrated that prenatal LPS injection increased nuclear factor (NF)-κB and TNF-α expression in the hearts of rat pups [18]. NF-κB is a downstream transcription factor in the TLR-mediated signaling pathway. The findings of these studies suggest that TLR4 is crucial in signaling cytokine production in the heart during endotoxic shock and maternal inflammation.

This study revealed high TLR4 expression in the hearts of the rats exposed to prenatal LPS, reared in either RA or O_2_, and treated with NS. This finding is consistent with those of recent studies suggesting that TLR4 activation contributes to the pathogenesis of chronic hypoxia-induced pulmonary hypertension and that TLR4-deficient mice are resistant to this condition [26, 27]. Ma et al. demonstrated that TLR4-deficient mice spontaneously developed pulmonary hypertension, which was not further enhanced by hypoxia [28]. The findings of these studies suggest that the role of TLR4 in mediating pulmonary hypertension is controversial and complex. LPS treatment increased β-MHC mRNA expression in the ventricular myocardium of rats and cultured rat cardiac myocytes [29, 30]. We observed an increase in the markers of right ventricular hypertrophy, pulmonary hypertension, and cardiac hypertrophy on postnatal day 14 in rats born to prenatal LPS-treated dams. These findings were supported by the increased TLR4 expression in the heart. Our results suggest that TLR4 mediates prenatal LPS-induced pulmonary hypertension and cardiac hypertrophy.

The beneficial effects of MSCs have been suggested to be predominantly influenced by paracrine effects [31, 32]. We observed that intratracheal MSC transplantation reduced right ventricular hypertrophy and pulmonary hypertension in prenatal LPS-treated rats. This paracrine effect of MSCs is analogous to that reported in a previous study, in which the intratracheal transplantation of stem cells attenuated hyperoxia-induced pulmonary hypertension in rats [13]. Additional studies are warranted to evaluate the optimal dose of MSCs in experimental pulmonary hypertension. In conclusion, intratracheal MSC transplantation protected cardiomyocytes from hypertrophy and reduced TLR4 expression, although we did not measure the specific factors. These results suggest that human MSCs protect cardiomyocytes by inhibiting TLR expression through a paracrine pathway.

## MATERIALS AND METHODS

### Animal model

Our study was approved by the Animal Care and Use Committee of Taipei Medical University. Time-dated pregnant Sprague–Dawley rats were housed in individual cages under a 12-h light–dark cycle, with ad libitum access to food and water. On gestational days 20 and 21, the rats received an intraperitoneal injection of LPS (0.5 mg/kg/day, *Escherichia coli* serotype 0111:B4; Sigma–Aldrich, St. Louis, MO, USA) dissolved in NS. The rat dams were allowed to deliver vaginally at term. Within 12 h of their birth, the pups were pooled, randomly redistributed to the mothers, and randomly assigned to either RA or O_2_ treatment. The pups in the O_2_ treatment subgroups were reared in an atmosphere of 85% O_2_ from postnatal days 1 to 14. The pups in the RA control subgroups were reared in normal RA for 14 days. The nursing mothers were rotated between the O_2_ treatment and RA control groups every 24 h to avoid O_2_ toxicity. An O_2_-rich atmosphere was maintained in a transparent 40 × 50 × 60-cm plexiglass chamber with a continuous supply of O_2_ at 4 L/min. O_2_ levels were monitored using a ProOx P110 monitor (BioSpherix; Redfield, NY, USA).

### Isolation of human MSCs

The study was approved by the ethics committee of the hospital, and written informed consent was obtained from all mothers before the study. Placental tissues were cut into small pieces (1–2 mm^3^) and digested with 10 U/mL collagenase, 2.5 U/mL dispase, and 0.05% trypsin–ethylenediaminetetraacetic acid for 90 min at 37°C. The tissue samples were subsequently collected in 15-mL tubes and centrifuged at 800 rpm for 5 min. The cell pellet fraction was resuspended in α-minimal essential medium (MEM; Invitrogen, Carlsbad, CA, USA) with 10%–15% fetal bovine serum (FBS, Invitrogen); 2 mM L-glutamine; 1 ng/mL basic fibroblast growth factor (FGF, PeproTech, Rocky Hill, NJ, USA); and 100 U/mL penicillin, 100 mg/mL streptomycin, and 0.25 mg/mL Fungizone (Invitrogen) and was subsequently plated in T75 flasks. The cells were passaged at approximately 70%–90% confluence, and the stem cells were maintained in α-MEM supplemented with 10% FBS, 2 mM L-glutamine, and 1 ng/mL basic FGF at 37°C under saturated humidity and 5% CO_2_. The MSCs were characterized by analyzing the expression of CD markers (CD44, CD73, CD90, CD105, CD11b, CD19, CD34, and CD45) and human leukocyte antigen–antigen D related through flow cytometry (BD Stemflow™ hMSC Analysis Kit, BD Biosciences, NC, USA). Furthermore, the capability of trilineage differentiation (osteocytes, chondrocytes, and adipocytes) and karyotypes yielded positive results.

### Human MSC transplantation

On postnatal day 5, human MSCs (3 × 10^5^ cells and 1 × 10^6^ cells) in 0.03 mL of NS were transplanted into the trachea of the pups by using a 30-gauge needle syringe. The administration route and MSC dose were based on the studies by van Haaften et al. and Chou et al., who have respectively demonstrated that the intratracheal injection of MSCs (1 × 10^5^, 3 × 10^5^, 1 × 10^6^ cells per animal) attenuates pulmonary hypertension induced by postnatal hyperoxia or prenatal LPS treatment in newborn rats [13, 18]. After the procedure, the pups were allowed to recover from the anesthesia and were returned to their dams. We examined five study groups: normal, LPS+RA+NS, LPS+O_2_+NS, LPS+O_2_+MSCs (3 × 10^5^ cells), and LPS+O_2_+MSCs (1 × 10^6^ cells). The normal group included rats that had received no treatment and were reared in the same environment as prenatal LPS-treated rats. The pups from each study group were strongly anesthetized with an overdose of gaseous isoflurane on postnatal day 14, and their body and heart weights were recorded. Immediately after death, their left lungs were ligated, and the right lungs were fixed by the tracheal instillation of 10% buffered formalin at a pressure of 25 cm H_2_O for 10 min.

### Western blotting analysis of β-MHC and TLR4

Cardiac tissues were homogenized, sonicated, and centrifuged at 13,000 rpm for 15 min at 4°C to remove cellular debris. Proteins (30 μg) were subjected to 12% sodium dodecyl sulfate polyacrylamide gel electrophoresis under reduced conditions and were electroblotted onto polyvinylidene difluoride membranes (Immobilon-P; Millipore, Bedford, MA, USA). After blocking with 5% nonfat dry milk, the membranes were incubated with TLR4 (SC-293072) and β-MHC (MYH7, SC-53090) antibodies (1:1,000; Santa Cruz Biotechnology, Dallas, TX, USA) or anti-β-actin (SC-47778), and they were subsequently incubated with horseradish peroxidase-conjugated antimouse IgG antibody (Pierce Biotechnology, Rockford, IL, USA). The protein bands were detected using a SuperSignal substrate (Pierce Biotechnology. A densitometric analysis was performed to measure the intensity of TLR4 and MYH7 expression and β-actin bands by using AIDA software. Data were normalized to β-actin for each animal.

### Right ventricular hypertrophy and pulmonary arterial remodeling

The lungs and hearts were removed and placed in a fixative at room temperature; 5-μm-thick lung sections were stained with hematoxylin and eosin for light microscopy analysis. Right ventricular hypertrophy was assessed by measuring the RV:LV thickness ratio. The MWT of vessels with an external diameter of 20–65 μm was measured. The wall thickness and external diameter were measured using Image-Pro Plus 6.0 (Media Cybernetics, Silver Spring, MD, USA). For each rat, 14–26 vessels were measured. The percentage medial thickness of an individual vessel was calculated using the following formula: (medial thickness × 2 × 100)/external diameter [33].

### Immunohistochemistry

Immunostaining was performed on the 5-μm-thick heart paraffin sections for immunoperoxidase visualization. After a routine deparaffinization step, heat-induced epitope retrieval was performed by immersing the slides in 0.01 M sodium citrate buffer (pH 6.0). Furthermore, to block the endogenous peroxidase activity and nonspecific antibody binding, the sections were first preincubated for 1 h at room temperature in 0.1 M phosphate-buffered saline containing 10% normal goat serum and 0.3% H_2_O_2_ and subsequently incubated for 20 h at 4°C with goat polyclonal anti-β-MHC (1:50; Santa Cruz Biotechnology Inc., Santa Cruz, CA, USA), mouse monoclonal anti-TLR4 (1:200; Abcam, Cambridge, MA, USA), and antihuman nuclei (1:100; Chemicon, Temecula, CA, USA) antibodies as primary antibodies. The sections were then treated for 1 h at 37°C with biotinylated rat antigoat IgG (1:200; Jackson ImmunoResearch Laboratories, West Grove, PA, USA) or goat antimouse IgG (1:200; Sigma-Aldrich). Following reactions with the reagents from the avidin–biotin complex kit (Vector, CA, USA), as per the manufacturer’s recommendations, the reaction products were visualized by incubating the sections with diaminobenzidine. Positively stained cells showed brown particles or clumps in the myocardial cell cytoplasm or nuclei of human MSCs. The mean optical density was used for a semiquantitative analysis. The positive staining area of each section under ×400 magnification was assessed using Image-Pro Plus 6.0.

### Statistical analysis

The data are presented as the mean ± standard deviation. Statistical analyses were performed using one-way analysis of variance with a Bonferroni post hoc test for multiple group comparisons. The differences were considered statistically significant at *P* < 0.05.
